# Anthralin—From Psoriasis Drug to Power Adjuvant

**DOI:** 10.3390/vaccines14070630

**Published:** 2026-07-18

**Authors:** Carolin Michael, Matthias Bros, Markus P. Radsak, Hansjörg Schild, Stephan Grabbe

**Affiliations:** 1 Department of Dermatology, University of Mainz Medical Center, Johannes Gutenberg University, 55131 Mainz, Germany; 2 Research Center for Immunotherapy, University of Mainz Medical Center, Johannes Gutenberg University, 55131 Mainz, Germany; 3 Department of Immunology, University of Mainz Medical Center, Johannes Gutenberg University, 55131 Mainz, Germany

**Keywords:** anthralin, dithranol, imiquimod, DIVA, cytotoxic T-cells, transcutaneous, immunization, vaccination

## Abstract

Anthralin has a long history as a topical treatment for psoriasis, where it reduces keratinocyte hyper-proliferation and effectively clears plaques. While it lowers inflammatory markers in psoriatic skin, it paradoxically induces inflammation in healthy skin through reactive oxygen species (ROS) and related pathways. However, its precise mechanism of action remains incompletely understood. Interestingly, the once undesirable pro-inflammatory effect in healthy skin may now represent a valuable adjuvant property for transcutaneous immunization (TCI). In particular, combining anthralin with the TLR7 agonist imiquimod (IMQ) elicits strong cytotoxic T-cell responses in pre-clinical studies. When paired with antigenic peptides that can penetrate the skin, this immunization approach is especially promising in the context of cancer therapy, given the central role of cytotoxic T-cells in tumor rejection. However, current evidence is largely derived from mouse models, but its efficacy and safety in humans remain to be established. This review therefore examines whether anthralin can be repurposed as a cutaneous adjuvant for transcutaneous immunization, and which mechanistic and translational constraints must be overcome before human application.

## 1. Introduction—From Antipsoriatic to Adjuvant

An extract from the tree *Vataireopsis araroba* (Aguiar) called Goa powder was used as early as 1876 to treat psoriasis. In 1912, a derivative of the compound chrysarobin found in Goa powder was synthesized (1,8-Dihydroxy-9-anthron). It is now known as anthralin as well as dithranol and is widely used to treat psoriasis due to its ability to reduce hyper-proliferation of keratinocytes. However, anthralin has side effects such as skin irritation and skin discoloration. Attempts to find a different derivative that has no side effects while retaining psoriasis treatment properties have failed so far [1].

However, the very side effect for psoriasis treatment may be a benefit for anthralin as a vaccine adjuvant. Swelling and reddening of the skin are signs of an inflammatory response at the treated area that leads to cytokine and chemokine release and to an accumulation of leukocytes [2]. Once immune cells infiltrate the skin, it is potentially prepared to receive a vaccine—a promising property of a vaccine adjuvant.

Current vaccine adjuvants primarily promote humoral immunity through an activation of the innate immunity followed by B-cell responses, which results in specific antibody production [3]. However, novel vaccine adjuvants are needed because some diseases cannot be prevented and treated by these immune responses. For example, it is now clear that immunological cancer treatment may be possible through the induction of strong, durable cytotoxic and memory T-cell responses [4].

This review therefore examines whether anthralin can be repurposed as a cutaneous adjuvant that promotes APC recruitment and conditioning for transcutaneous immunization, and which mechanistic and translational constraints must be overcome before human application.

## 2. Classical Role of Anthralin in Psoriasis

For psoriasis treatment, anthralin is most widely applied in a Vaseline base made by local pharmacies. Varying dosages between 0.03 and 2% are common. The inflammatory side effects of anthralin decrease with repetitive topical applications to the same skin site. Therefore, application regimens either utilize extended contact times or increased concentrations of anthralin, but responses are highly variable between patients [5,6].

Anthralin exerts only local effects, as it barely reaches the dermis in healthy skin and penetrates a little deeper into the dermis in psoriatic skin, where it is rapidly metabolized within 1–2 h [7]. One of the side effects of anthralin is a brown discoloration of the skin after topical treatment, not caused by melanin. Instead, oxidized forms of anthralin have a brown appearance and remain in the skin, discoloring it. This leads to everyday problems because clothing or furniture that come into contact with the discolored skin becomes stained [8,9].

In attempts to reduce side effects and to increase storage stability, several novel, anthralin-based formulations have been developed. Salicylic acid has been widely combined with anthralin in topical formulations, not only to enhance de-scaling of psoriatic plaques but also to increase skin penetration and aid chemical stability [10]. Anthralin was also formulated as liposomal, ethosomal, and dendrimer entrapped microsponge gels, and micro-encapsulated in crystalline monoglycerides known as Micanol^®^. However, none of these new formulations has so far out-competed anthralin in a Vaseline base in clinical practice [11,12,13,14].

## 3. Mechanistic Insights

Since anthralin is a common psoriasis drug, mechanistic research has been performed on various primary or immortalized cell cultures, healthy and psoriatic human skin, and untreated or psoriasis modeled mouse skin. In the following, we discuss the various effects of anthralin, reported to date.

### 3.1. Biochemical Pathways

**Figure 1:**
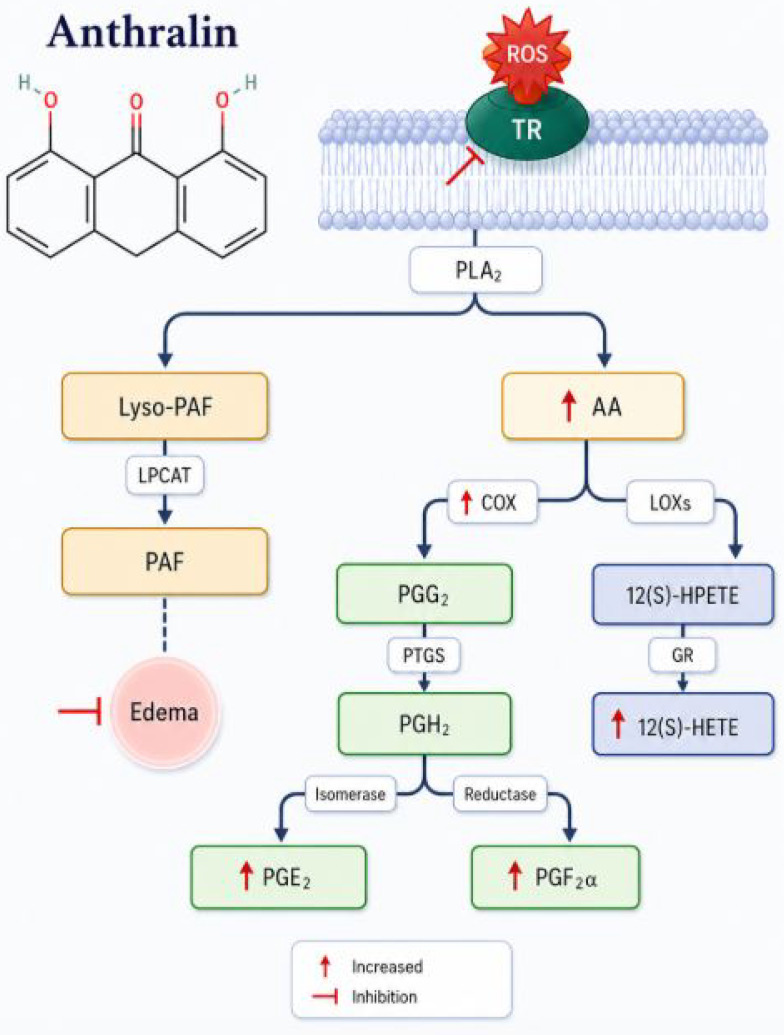
ROS pathways influenced by anthralin and their relationships.

Thioredoxin reductase (TR) resides at the plasma membrane of various cell types including keratinocytes (Figure 1). By catalyzing the reduction of free radicals, TR can protect cells from oxidative stress. Its increased activity in psoriatic vs. healthy skin indicates that psoriatic skin contains more free radicals than uninvolved skin. TR also plays a role in anthralin’s mode of action. Anthralin binds to and inactivates TR’s active site, effectively incapacitating the enzyme [15]. In a parallel way, anthralin causes lipid peroxidation of cell membrane components [16].

Cyclooxygenase (COX) converts arachidonic acid (AA) into prostaglandins (PGE2 and PGF2α). AA, COX2, and PGE2/PGF2α are elevated after anthralin treatment (Figure 1) [17,18,19,20]. The release of AA only partially accounts for the biologic effects of anthralin since topical application of AA causes acute but short-lived skin inflammation. However, anthralin causes a slowly increasing, but more intense and longer-lasting inflammatory reaction. Co-application of AA with anthralin intensifies the inflammatory response at early time points. Later on, however, the reaction intensity is far below the levels of anthralin application alone. Specifically, at 6 h, erythema (redness of the skin) and edema (swelling of the skin) more than double, whereas at 72 h, co-application cuts these levels to about half of the anthralin treatment alone [21]. In contrast to healthy skin, it may be due to psoriasis-associated elevated levels of AA that anthralin does not cause erythema and edema there.

12-hydroxyeicosatetraenoicacid (12(S)-HETE) is a bioactive lipid ligand and product of arachidonic acid that is elevated in psoriasis as well as after anthralin treatment (Figure 1) [20]. In contrast, anthralin was found to reduce the number of 12(S)-HETE receptors while receptor affinity remains constant [22,23]. Interestingly, 12(S)-HETE increases Ca^2+^, a trigger for oxidative stress, which is followed by TR production. It is possible that this represents a positive feedback loop for oxidative stress during anthralin treatment since anthralin blocks TR.

The application of peroxyl radical scavengers (α-tocopherol, butylated hydroxy-anisole, retinol acetate, retinol palmitate, and PEG-SOD) dampens erythema and edema significantly, while the scavengers of hydroxyl radicals mannitol and N-acetyl cysteine had no such effects. Additionally, the non-classical radical scavenger tert-butylphenylnitrone was found to reduce the amount of free radicals present in the skin after anthralin application [24,25,26]. However, it remains unclear which type of free radical species are induced by anthralin and through which pathway they arise. Unfortunately, the effect of free radical scavengers in combination with anthralin on psoriatic human skin has not been studied yet.

Platelet-activating factor (PAF) activates intracellular calcium and protein kinase C, which in turn stimulates NADPH oxidase and leads to increased production of superoxide and other ROS species [27]. Some PAF antagonists minimize anthralin-induced edema (Figure 1) [28,29].

Future research should delineate which molecular steps within the TR–PLA_2_–AA–PAF axis are directly controlled by anthralin-derived ROS and which are secondary responses. A systematic analysis concerning the contribution of distinct PLA_2_, COX, LOX and LPCAT isoforms as well as the relevant PAF and 12(S)-HETE receptors to anthralin’s mechanism of action may give much insight here. Time-resolved lipidomics and ROS-species profiling after selective inhibition of TR, PLA_2_, COX, LOXs, and NADPH oxidase would help to clarify the sequence of events linking TR inhibition to AA release, PGE_2_/PGF_2_α formation, 12(S)-HETE production and PAF-driven edema. Moreover, it remains unexplored whether restoring redox balance with defined radical scavengers, or modulating Ca^2+^ signaling downstream of 12(S)-HETE and PAF, can uncouple the desired antipsoriatic or adjuvant effects of anthralin from its local inflammatory effects. Carefully controlled in vivo studies in patients and advanced 3D skin models should therefore test combinations of anthralin with pathway-specific inhibitors (e.g., PAF antagonists, LOX/COX inhibitors, NADPH oxidase blockers) and radical scavengers, with parallel readouts of TR activity, eicosanoid profiles and clinical inflammation, to validate and refine the proposed feedback loops highlighted in the pathway model.

### 3.2. Cellular Effects

It is established that anthralin accumulates in mitochondria, oxidizes within 1–2 h, and causes apoptosis in keratinocytes [30,31,32]. Apoptotic changes are associated with dissipation of mitochondrial membrane potential, cytochrome *c* release, caspase-3 activation, phosphatidylserine externalization, and finally, morphological changes associated with apoptosis. Anthralin acts by disrupting the respiratory chain through the ubiquinone pool. Specifically, there is evidence that the modulation on the electron flow through the respiratory chain is facilitated by the ubisemiquinone anion [32].

Anthralin inhibits the proliferation of keratinocytes in psoriasis plaques [33]. The clinical response of anthralin on psoriatic skin features up-regulation of Cytokeratin 16, which leads to increased differentiation of keratinocytes. This ultimately results in decreased skin thickness [5]. However, when compared to other topical irritants, there is no correlation between the severity of the inflammatory reaction and proliferative rate of keratinocytes, demonstrating that the inflammatory reaction and the proliferation inhibition may act in two distinct pathways [34].

Interestingly, anthralin effectively reduces neutrophil infiltration only in human psoriatic plaques, thereby targeting neutrophilic motility and chemotaxis, a hallmark of psoriasis. Neutrophil chemotaxis and random migration are inhibited through lower *f*MLP binding to formyl peptide receptor (FPR) receptors. These effects pair with keratinocyte apoptosis and inhibition of chemokine expression, such as CXCL8 down-regulation via IL-36 inhibition. Consequently, anthralin reduces psoriatic epidermal hyperplasia within a few weeks. In contrast to psoriatic skin sites, where it exerts antiproliferative and anti-inflammatory effects, anthralin triggers an inflammatory reaction in perilesional, clinically normal skin [5,35].

Epidermal growth factor (EGF) and transforming growth factor alpha (TGF-α) are overexpressed in psoriatic skin. Therefore, a reasonable hypothesis is that lowering EGF and TGF-α to normal levels might be beneficial for psoriasis treatment. Anthralin decreases TGF-α as well as EGF-receptor (EGFR) binding in keratinocytes in a dose-dependent manner [33,36]. Additionally, the transcriptional regulator inhibitor of DNA binding 1 (Id1) was found to be important for epidermal progenitor cell proliferation [37]. Anthralin down-regulates Id1 mRNA after a 4 h treatment procedure in HaCaT keratinocyte cell culture, and Id1 protein levels are not affected at that time, but this might very well be the case at a later time point [38]. Furthermore, Human Epidermal Growth Factor Receptor 2 (HER2; ErbB2) was found to be down-regulated by anthralin, although this was found in oral cancer cell lines and needs to be verified in skin or skin cells [39]. Interestingly, Id1 as well as HER2 are involved in the EGFR pathway. Hence, the EGFR axis may be a relevant axis of anthralin treatment.

Future studies should dissect in detail how anthralin-induced mitochondrial dysfunction and apoptosis in keratinocytes are coupled to changes in proliferation. Furthermore, the role of an intact respiratory chain and interaction with the ubiquinone pool concerning differentiation markers and long-term tissue remodeling should be studied in vivo. Time-resolved experiments in primary human keratinocytes and advanced 3D skin models, combining mitochondrial bioenergetics and single-cell apoptosis readouts with downstream measurements of EGFR/TGF-α signaling, Id1 and HER2 expression, and chemokine production, could be used to map this axis more precisely. In addition, the selective effects of anthralin on neutrophil migration and FPR signaling, and their integration with IL-36/CXCL8 pathways and keratinocyte mitochondrial stress responses, remain to be clarified using co-culture and in vivo models. Finally, confirmation of anthralin’s effects on Id1 and HER2 at the protein level in skin, alongside functional perturbation of these nodes, would be crucial to validate an EGFR–Id1–HER2–mitochondrial mode-of-action axis.

### 3.3. Things We Know Anthralin Does Not Affect: Important Null Data

Some plausible pathways for the accumulation, oxidation and apoptosis-inducing function of anthralin have been found to be independent of mitochondrial function. Mitochondrial anthralin oxidation is not ATP-dependent, and the ATP-generating electron transport chain is not involved in a relevant way either. Furthermore, there is evidence that the increase in free radicals after anthralin treatment is not facilitated by enhanced autoxidation of anthralin by mitochondrial/microsomal proteins [30].

The aryl hydrocarbon receptor (AhR) is a transcription factor that is highly expressed in barrier tissues including the skin, gut, and lung. Because these organs are central to immune defense, it is not surprising that AhR plays a crucial role in immune regulation and the maintenance of immune homeostasis [40]. Notably, AhR activation has been shown to dampen psoriasis symptoms [41]. Moreover, coal tar—one of the oldest therapeutic treatments for psoriasis—mediates its anti-psoriatic effects through modulation of AhR signaling [42]. In addition, 6-Formylindolo [3,2-b]carbazole (FICZ), a photoproduct generated during UV light therapy, as applied in psoriasis treatment, has been identified as an endogenous ligand of AhR [43]. Finally, anthralin’s chemical structure, including three benzene rings, matches potential ligands of AhR [40]. To test whether anthralin functions via the AhR pathway, AhR-deficient mice were treated topically with anthralin. However, AhR deficiency did not modulate the response to anthralin treatment [5]. Specifically, treated dorsal and ear skin samples were embedded in paraffin and analyzed for epidermal thickness and inflammatory infiltrate. Compared to controls, neither measure rendered significant results, suggesting that anthralin does not act via AhR activation [44].

Calmodulin is a sensory, calcium-binding protein in the skin that is known to be elevated in psoriasis. Therefore, it was tested if anthralin inhibits calmodulin in vitro. Anthralin was in fact found to inhibit calmodulin, however, its clinically non-relevant oxidized versions also inhibited calmodulin in vitro [45]. Calmodulin may therefore not be a relevant target of anthralin.

The CD40/CD40L axis enables crosstalk between T-cells and APCs. It is key for signaling inflammatory responses as well as cellular immunity against infection and cancer [46]. Bcl-2 is known to act in an anti-apoptotic way and thereby aids cell survival [47]. Psoriasis patients have elevated serum-levels of CD40/CD40L, and surprisingly, these levels remain high after successful anthralin treatment and substantial plaque reduction. In contrast, no differences in serum levels of Bcl-2 were documented [48]. However, CD40/CD40L have been found to be up-regulated and Bcl-2 down-regulated in psoriatic skin, while studies on the effect of anthralin on healthy skin levels are lacking [49,50].

An important next step is to better define the non-mitochondrial redox pathways responsible for anthralin oxidation and ROS generation by pinpointing the relevant cytosolic and membrane-bound oxidoreductases and quantifying their contribution relative to mitochondrial sources. The inconclusive findings for candidate targets such as AhR, calmodulin, and systemic CD40/CD40L and Bcl-2 levels illustrate the importance to report such data, as they substantially narrow the spectrum of plausible pathways and help prevent redundant lines of investigation. Continued publication of such null data is essential to refine models of anthralin’s mode of action and to focus future work on the most promising signaling axes.

## 4. Anthralin as an Immune Modulator

As psoriasis is a T-cell-mediated autoimmune disease that responds to treatment with common immunosuppressants such as corticosteroids and cyclosporine, it appears counter-intuitive that a substance that is effective against psoriasis might be suitable as an immune adjuvant and may enhance T-cell-mediated immune responses. However, as mentioned earlier, anthralin also has pro-inflammatory properties, which long have been regarded as nothing more than a pesky side-effect. It now becomes clear that this feature might be beneficial in some applications. Therefore, much more is to learn about the immune-modulatory function of anthralin, a very interesting field of current investigation. In the following, the effects of anthralin on healthy, not psoriatic skin or cell cultures are discussed. Intriguingly, the effects of anthralin on psoriatic skin can be the exact opposite of those on healthy skin, some of which cases are discussed in Section 2 of this review.

### 4.1. Inflammation (Cytokine and Chemokine Signatures, AMPs)

Generally, ROS play an instrumental role in the inflammatory response, serving as key signaling molecules. ROS activate pathways that amplify pro-inflammatory cytokine and chemokine production, critical for immune cell recruitment [51]. Free radical scavengers dampen the inflammatory skin reactions erythema and edema after anthralin treatment significantly [24]. This confirms that free radical formation triggers inflammatory processes.

**Figure 2:**
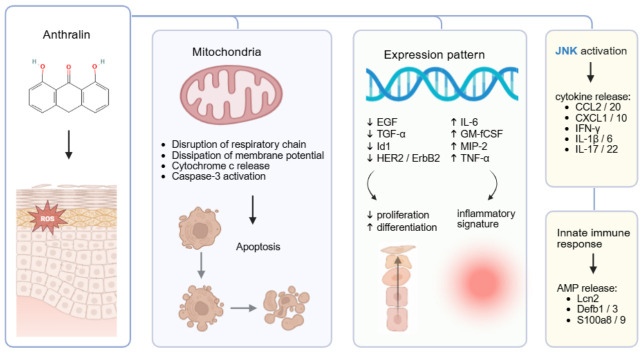
Anthralin-induced cellular events and downstream effects.

The levels of mRNA encoding the pro-inflammatory cytokines IL-6, GM-fCSF, MIP-2, and TNF-α are elevated upon anthralin treatment (Figure 2). In response to anthralin and α-tocopherol in combination, MIP-2 and TNF-α are transcribed at lower levels than upon treatment with anthralin alone, but still exceed untreated controls. α-tocopherol does not change the expression levels of IL-6 and GM-CSF when combined with anthralin [25].

Anthralin activates the JNK pathway in a ROS-dependent manner, which is in line with the general observation that the JNK pathway is generally activated by stress signals such as ROS and in turn regulates inflammation, apoptosis, and proliferation. Ultimately, the JNK pathway promotes chemokine and cytokine release, including CCL2, CCL20, CXCL1, CXCL10, IL17, IFN-γ, IL-1β, IL-6, and IL-22. Therefore, anthralin-induced ROS is an essential upstream mediator of anthralin-induced JNK activity [16,52,53].

Anthralin induces an anti-microbial response in the skin indicated by up-regulation of several anti-microbial peptides (AMPs). Specifically, the AMPs Lcn2, Defb1, Defb3, S100a8, and S100a9 are increased, suggesting that anthralin activates innate cutaneous defense pathways that may contribute to its immunomodulatory effects [54].

### 4.2. Myeloid Cells: Recruitment, Differentiation, and Disruption

The expression of adhesion molecules E-selectin (also known as ELAM-1 or CD62E), VCAM-l, and ICAM-1 by microvascular endothelial cells is increased after anthralin treatment [55]. These promote leukocyte extravasation, enabling immune cells to exit the blood stream and enter tissues, boosting local immune cell influx.

Keratinocytes can up-regulate MHC class II expression in response to various inflammatory stimuli. It is therefore unsurprising that anthralin treatment enhances MHC class II expression on keratinocytes in skin samples. Therefore, keratinocytes may act as non-classical APCs following anthralin treatment, although functional studies have not been performed yet [56,57].

Anthralin application leads to an early influx of immune cells into the skin, including T-helper cells (CD4^+^), cytotoxic T-cells (CD8^+^), and dermal dendritic cells (DCs; CD11c^+^). However, this effect is not unique to anthralin but a rather comparatively general reaction to numerous irritants [55,58]. Epidermal Langerhans cells (LCs, CD1^+^; Leu6/CD1a^+^) were found to be diminished 48 h after anthralin treatment [59,60]. The morphology of the remaining LCs featured few dendrites. Electron micrographs additionally revealed that LCs display disrupted cristae within swollen mitochondria 8 h after anthralin treatment. Surrounding keratinocytes appeared normal at that time point [59]. Given this information, anthralin stressors induce one of two epidermal LC fates: death/apoptosis or activation.

Monocyte recruitment and genes expressing monocyte differentiation markers are up-regulated in the skin after anthralin treatment. This is underscored by the presence of monocyte-derived DCs (moDCs) and macrophages in the treated skin areas as well as in the draining lymph nodes [2]. Anthralin-induced APC activation may extend beyond classical maturation markers and involve additional co-signaling pathways that shape downstream T-cell responses. In dendritic cells, antigen-presenting capacity is linked not only to increased expression of MHC-II and co-stimulatory molecules but also to migratory competence and cytokine production. It will therefore be important to determine whether anthralin influences the broader balance between co-stimulatory and co-inhibitory signals at the APC–T-cell interface. A more detailed characterization of these markers could refine the current view of anthralin as a cutaneous adjuvant and help define which APC states are most relevant for efficient T-cell priming [61].

Interestingly, macrophages in the draining lymph node exhibit increased expression of the activation markers CD80 and CD86 upon anthralin treatment. Somewhat surprisingly, moDCs show no such activation. Assessment of metabolic activity shows that macrophages also exhibit metabolic changes upon anthralin treatment. Specifically, the oxygen consumption rate decreases, indicating that mitochondrial respiration is impaired. Additionally, the basal rate of glycolysis, as measured by assessment of the extracellular acidification rate, increases. This data indicates that anthralin drives macrophages towards an anaerobic glycolysis metabolism [2].

Taken together, anthralin exerts selective myeloid cell regulation, promoting monocyte recruitment into the skin and differentiation into moDCs or macrophages. Early LC emigration and up-regulation of adhesion molecules in endothelial cells constitute part of this distinctive immune response.

Future work should systematically dissect how anthralin-induced ROS, JNK activation, and AMP induction translate into a qualitatively distinct “adjuvant-type” inflammatory milieu, ideally using time-resolved cytokine/chemokine and AMP profiling in skin models and in vivo. In particular, it is important to define the cellular sources of these signaling molecules to clarify how endothelial adhesion molecule up-regulation, keratinocyte MHC class II expression, and the divergent fates of Langerhans cells versus monocyte-derived DCs work together to initiate antigen presentation and T-cell priming. Functional assays of antigen uptake and presentation should reveal under which dosing or formulation conditions these responses can be harnessed with minimal side effects.

## 5. DIVA

The TCI strategy using anthralin and IMQ as complementary acting adjuvants is termed DIVA (**D**ithranol/**I**MQ based **VA**ccination). So far, only OVA/SIINFEKL/OT-II mouse models have been utilized for the investigations presented in this chapter.

### 5.1. DIVA Adjuvants

#### 5.1.1. Anthralin

Current knowledge about the immunomodulating properties of anthralin are highlighted in Section 4 of this review. The skin is generally regarded as a tolerogenic organ in the steady state, where exposure to antigen in the absence of danger signals tends to favor regulatory or non-responsive T-cell outcomes rather than productive immunity [62]. As with other vaccine adjuvants, pro-inflammatory cues are therefore required to shift this balance toward robust effector and memory responses [3]. Anthralin was specifically selected for DIVA because of its ability to induce a controlled local inflammatory milieu at the site of antigen application. DIVA is the first immunization approach that utilizes anthralin as an adjuvant [2,63].

#### 5.1.2. IMQ

IMQ is an immunomodulating agent, currently used in the clinic as topical treatment for basal cell carcinoma, actinic keratoses and anogenital warts. IMQ is known to act as a TLR7/8 agonist activating antigen presenting cells [64]. For that reason, TLR7/8 agonists such as IMQ are under investigation as possible vaccine adjuvants. The knowledge presented in the following chapter originates from mouse models.

##### Strengths and Shortcomings of IMQ-Only TCI

A TCI with IMQ (Aldara^®^) in combined application with antigen peptides induces an increase in cytotoxic T-cell (CTL) numbers, their maturation and activation, as confirmed by IFN-γ production upon re-stimulation and specific lysis of peptide-loaded target cells. This reaction is of short-lived nature though, as the frequency of CTLs rapidly declines after its peak 8 days after TCI. In addition, the specific lysis capacity of CTLs also reduces from over 90% at 7 days to about 10% at 35 days post-TCI, indicating a lack of memory formation. Consequently, IMQ TCI does not provide full prophylactic or therapeutic protection in OVA/SIINFEKL tumor immunization studies [65,66,67].

A better understanding of the principal mechanisms involved in IMQ TCI may enable a directed optimization, possibly by increasing immune response mediators and bypassing or blocking suppressive factors. An immunosuppressive factor known from cancer research are regulatory T-cells (T_reg_), which limit APC activation via IL-10 production among other mechanisms. In fact, T_reg_ as well as IL-10 impair the efficacy of IMQ TCI, while depletion of T_reg_ leads to an increased percentage of TCI-induced CTLs, and IL-10 deficiency improves various metrics of TCI efficacy. However, neither T_reg_ nor B-cells are the cellular source of IL-10 in this setting, requiring further investigation [68]. Furthermore, an involvement of mast cells in the immune response to IMQ has been established. Mast cells become activated by IMQ through TLR7 agonism and release both IL-1β and TNF-α as a result. LC emigration is dependent on mast cells and mediated by mast-cell-derived IL-1 but not TNF-α. Priming of CTLs following IMQ TCI is also mast cell-dependent [69].

To improve IMQ TCI, new formulations were developed and investigated regarding their adjuvant efficacy. Aldara^®^ contains IMQ in a dissolved state. An IMQ emulsion gel (IMI-Gel) shows comparable adjuvant properties as dissolved-state IMQ. Although principally suitable, there is no improvement upon the already existing formulation [70]. A solid IMQ nanoemulsion (IMI-Sol) shows superior adjuvant potential as reflected by an increased frequency of antigen-specific CTL, specific lysis of peptide-loaded target cells, and IFN-γ production upon re-stimulation. However, this formulation also contains an appreciable amount of α-tocopherol, a free radical scavenger used as a preservative for the lipid component squalene [63,71,72]. The α-tocopherol content may be of concern when combined with anthralin, as this free radical scavenger dampens the inflammatory capacity of anthralin (discussed in Section 3.1). Taking both points together, this will be further discussed in Section 6.

##### Combination with Other Adjuvants

Given the need for a prolonged immune response with memory T-cell formation, several combination approaches were explored with the hope of achieving such an effect. Depending on availability, either IMQ or IMI-Sol TCI was examined. All tumor studies presented here describe the effects of the well-established model antigen system OVA/SIINFEKL. Some combination adjuvants are injected, meaning these approaches are no longer solely transcutaneous.

CD40 agonists are investigated in the broader cancer treatment community because of their capacity to activate APCs. Specifically, CD40-mAb cross-links CD40 on APCs, imitating ligation with CD40L and thereby promoting maturation [73]. Injection of CD40-mAb enhances, but does not provide, full tumor protection in combination with either IMQ or IMI-Sol [67,74]. A similar picture paints itself for the compound cyclophosphamide (Cy), which is known to inhibit T_reg_ in tumor settings. As mentioned in Section 5.1, T_reg_ show a suppressive function for IMQ TCI. To determine whether Cy may therefore strengthen the immune response to IMQ TCI, the two were tested in combination. However, IMQ TCI with Cy injection only lead to modest increases in DC activity and no improvement in tumor protection when compared to IMQ TCI alone [75].

To enhance IMQ TCI-induced mast cell activation, the transient receptor potential melastatin-4 (TRPM4) agonist 9-phenanthrol (9-phe) was applied topically as a co-adjuvant. The addition of 9-phe increases de-granulation of mast cells, although surprisingly not in a TRPM4-dependent manner. The combination treatment leads to an increase in survival time in a prophylactic tumor setting but does not provide full tumor protection [69,76].

### 5.2. The DIVA Protocol Design

**Figure 3:**
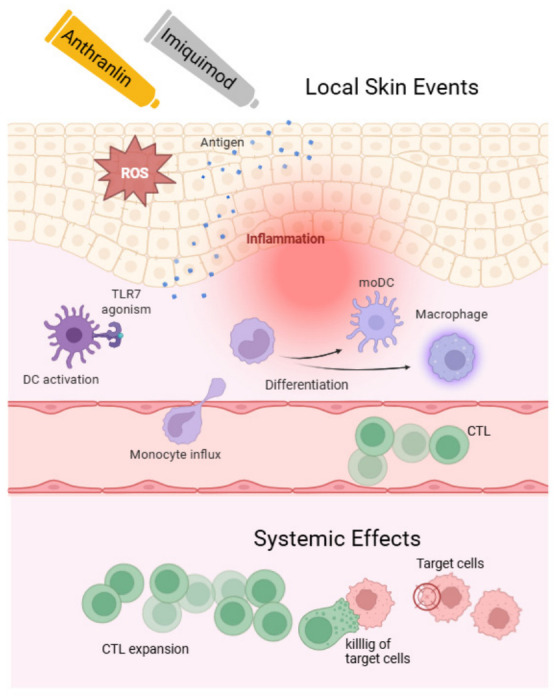
Effects of DIVA that may contribute to the adjuvant effect.

DIVA was developed with anthralin in a Vaseline base and IMQ as an IMI-Sol formulation [71]. According to the DIVA protocol, mouse ears are sequentially treated with anthralin, IMQ, and antigen peptides (Figure 3). Repeating the protocol one week later is termed DIVA^2^ and boots vaccination efficacy. A third application renders no improvement. Reducing the treated skin area to only one ear shows a trend towards a reduced efficacy, indicating that treatment of both ears is the necessary area needed. The amount of anthralin needed for efficient DIVA is far below the doses applied for psoriasis models [2,63].

### 5.3. Immune Cell Response Profiles

In mice, DIVA induces a significant increase in primary and memory CTL numbers and their activation, as confirmed by IFN-γ production upon re-stimulation and specific lysis of peptide-loaded target cells. The further optimized DIVA^2^ indicates that repeating the protocol further boosts CTL responses dramatically. Primary CTL numbers remain constant until they show only a slight decrease after 49 days of initial vaccination. Memory CTLs display good effector function, indicating a long-lasting and potent immune response [2,63].

To delineate through which mechanisms DIVA unfolds its potential, several hypotheses were tested. Since IMQ is a TLR7 agonist, it was of interest if agonistic effects to a specific cell type uniquely shape DIVA efficacy. It appears that TLR7-dependent activation of DCs is more important for DIVA efficacy than TLR7-dependent activation of PMNs or monocytes/macrophages. Furthermore, depletion of CD4^+^ T-cells during DIVA vaccination leads to a diminished CTL response, indicating that DIVA efficacy is CD4^+^ T-cell-dependent [2].

Further investigation reveals that anthralin causes a sharp increase in monocytes, monocyte-derived DCs (moDCs), and macrophages in the treated skin area. The draining lymph nodes also show an influx of moDCs and macrophages after anthralin treatment. Surprisingly, when comparing anthralin to DIVA, the amounts of moDCs and macrophages remain constant, but DIVA enhances their activation states. This effect highlights the synergistic effects of anthralin and IMQ, where anthralin may trigger early monocyte influx and IMQ activates immune cells [2].

As discussed in Section 3.1, anthralin is known to induce ROS, and the free radical scavenger α-tocopherol reduces inflammatory markers such as erythema and edema [24]. Anthralin’s effect on monocyte recruitment to the skin proves to be ROS-dependent as well, because co-application with α-tocopherol (although injected, not topical) is followed by only minimal monocyte influx. Thus, it appears that monocyte recruitment is vital for DIVA efficacy because co-application with α-tocopherol induces an immune response of diminished potency. Further substantiating this hypothesis, monocyte depletion through the MC-21 antibody reduces DIVA’s immunization effects [2].

Some immune cell response profiles have been studied in tumor microenvironment settings, which are discussed in the next chapter.

### 5.4. Anthralin as an Immune Adjuvant in Murine Tumor and Virus Vaccination Models

During TCI with anthralin-containing formulations, local immune responses in the skin play a central role. Infiltrating monocytes are crucial for the adjuvant effect of DIVA formulations at the site of application [2]. These cells may contribute to the initiation of a potent and long-lasting CTL immune response by enhancing local antigen presentation and inflammation.

Prophylactic DIVA^2^ vaccination fully prevents tumor development in the MC38mOVA model [63]. However, treatment of established tumors with the same model does not achieve complete tumor protection. Although initial tumor control is observed, growth eventually resumes, paralleling untreated controls. This “immune evasion phase” correlates with the emergence of immunosuppressive CCR2^+^ monocytes within the tumor microenvironment and the loss of CTL-mediated tumor control. Depletion of immunosuppressive CCR2^+^ monocytes by the anti-CCR2 (MC-21) antibody partially restores tumor suppression [77]. Additionally, DIVA vaccination markedly reduces the viral load in prophylactic vaccinia virus infections [2], indicating its efficacy to mount strong antiviral immunity.

Given the dual role of monocytes—essential for initial immune priming but later contributing to tumor immune evasion—combining topical DIVA-based TCI with systemic treatment targeting CCR2^+^ monocytes may enhance therapeutic efficacy. While no longer solely needle-free, this integrated approach could optimize immune activation while minimizing immunosuppressive feedback within the tumor microenvironment.

### 5.5. Limitations and Open Questions

Several important questions remain regarding how DIVA achieves the potent yet context-dependent adjuvant effects. Building on the observation that anthralin-driven ROS and IMQ-mediated TLR7 agonism jointly shape monocyte influx, moDC differentiation, and CTL priming, it is still unclear which monocyte and DC subsets are indispensable, how their activation states change over time, and how far these dynamics can be modified by dose, timing, and antigen format.

Furthermore, the potential of DIVA as an immunotherapy or vaccination protocol for human tumors still needs to be investigated. Experimental data on metastasis prevention and on prevention of spontaneously arising tumors in murine models is still lacking, and so is data on tumor-relevant antigens other than the well-established OVA/SIINFEKL/OT-II model in tumor settings. Available data for infectious diseases is limited to a vaccina model in a preventive setting. Moreover, although all components of DIVA are approved for clinical application, its combination for vaccine purposes has not been assessed in humans yet.

The intact human skin barrier prevents anything larger than roughly 500 Dalton from passing when applied topically [78]. However, the well-established model antigen SIINFEKL has a weight of 963 Dalton and still elicits profound immune responses when applied topically and with adjuvants, although this was found in mice [67,68,69,70,71]. It is unclear whether anthralin and IMQ enable enhanced penetration through the skin. However, transcutaneous uptake of this large peptide is suggested to be very efficient, as there are no detectable differences in immune response between intradermal and topical application of SIINFEKL antigen in an IMQ TCI model [66]. Nevertheless, the amount of SIINFEKL that is applied topically does matter, as the immune response followed by 100 µg of peptide is superior to 30 µg of peptide [63]. SIINFEKL is slightly hydrophobic, which aids skin penetration. Hydrophilic peptides may not work as well, although this is not established yet. The maximum size of antigenic peptides for DIVA^2^ also remains unclear. Other supporting mechanisms for peptide uptake may be combined with DIVA to deliver problematic peptides. For example, tape stripping or occlusive dressings have been successfully applied to overcome the stratum corneum as a mechanical barrier for polar or macromolecular substances in humans [79,80]. Should insufficient peptide uptake be a hurdle in the future, adding such a skin preparation step represents a possible solution.

## 6. Translational Challenges—From Mouse Skin to Human Application

### 6.1. Mouse Versus Human: What Matters for DIVA?

DIVA has been studied in mouse models so far but there are fundamental differences between mouse skin and the human target species. Specifically, the loose mouse skin is about four times thinner than human skin, which is firmly attached to the underlying tissue. In contrast to the relatively sparse human body hair, mice have a largely uniform fur coat with closely spaced hair follicles over most of the skin surface. This higher follicle density in mice suggests that follicle-associated pathways may contribute more to the uptake of topically applied substances than in many human non-scalp regions. At the same time, studies in human skin show that hair follicles function as efficient reservoirs for topically applied compounds and can contribute measurably to overall cutaneous absorption. These species differences in follicle number and organization therefore directly affect how results from mouse skin models are translated to human applications in topical drug delivery and transcutaneous immunization [81,82].

While there are additional differences in the immune system of both species, they share many cell types involved in immune responses. Mouse T-cells have a much shorter half-life than human T-cells [83]. For DIVA, this means that memory T-cells might last much longer in humans than measured in mice. Inflammatory cytokines and their receptors differ between humans and mice [84]. Since DIVA relies on monocyte attraction and differentiation [2], for which cytokine release and receptor binding is crucial, these differences may be of importance. Furthermore, EGF-R is known to be expressed in human but not in mouse skin and anthralin is known to impair EGF-R binding in keratinocytes [33,36,84]. Thus, mouse models cannot precisely predict the effects of DIVA on human skin.

The available DIVA data were obtained mainly in normal skin, but translational application will likely need to consider patients with pre-existing skin inflammation, barrier defects, or chronic dermatologic disease. In such settings, baseline immune activation, tissue permeability, cytokine composition, and cellular recruitment may differ substantially from those in healthy skin, with potential consequences for both immunogenicity and local tolerability. While inflamed skin could in principle enhance antigen access and APC activation, it may also render the response less predictable and increase reactogenicity. Thus, the performance of DIVA in inflamed or diseased skin represents an important open question for future translational studies.

Moreover, mice have a total of 50–80 cm^2^ skin [85]. About 3 cm^2^ are subject to DIVA treatment, roughly 4–6% of the skin area. Humans have a total of 1.6–1.8 m^2^ skin [86]. If the relative size of skin area needs to be the same, an area between 25 cm × 25 cm and 33 cm × 33 cm (625–1089 cm^2^) would need to receive treatment. This is a rather large area and certainly represents a challenge for translation to humans. However, it is unknown whether a proportionally large skin area needs to be treated with DIVA to induce comparable immune responses also in man. It might be argued that the option to treat large skin regions also provides an opportunity to further increase vaccine-induced immune responses beyond the level achieved in mice. Systematic variation in treated surface area and anthralin/IMQ dose in such models could clarify whether a fixed minimal “critical area” is required for robust responses in humans, or whether higher local antigen and adjuvant densities can compensate for smaller fields of application. In parallel, mechanistic work should dissect how human-specific features such as cutaneous EGFR expression and distinct cytokine–receptor networks shape DIVA-induced immunity, thereby informing rational dose and area scaling for early-phase clinical trials.

### 6.2. Reactogenicity, Tolerability and Patient Acceptability

Anthralin induces an inflammatory reaction in healthy skin, causing swelling, reddening, and brown staining of treated areas, although the concentrations used for TCI are lower and are therefore expected to cause milder side effects. DIVA uses anthralin in a dosage of 0.3 µg/mg ointment, whereas psoriasis treatments require between 0.3 and 20 µg/mg. The dosage needed for TCI is therefore far below concentrations determined as safe, although this dosage needs to be confirmed for human use. The low dosage is expected to be better tolerated because the inflammatory skin response is dose-dependent [9,24,25,26,63]. Side effects of IMQ treatment include reddening of the skin area, as well as itching and burning sensations [64]. Taken together, DIVA TCI poses some potentially unpleasant side effects, and the combination of the two topicals has not been evaluated in humans. However, especially considering therapeutic cancer immunization, some side effects can be considered acceptable.

### 6.3. Regulatory and Practical Hurdles

Only a small number of adjuvants are currently licensed for clinical use. Medical regulatory agencies consider adjuvants as a sensitive component of vaccines. Current guidance emphasizes that each new adjuvant–antigen combination must demonstrate a clear immunological benefit without a considerable increase in side effects. In both FDA and EMA frameworks, adjuvants are not licensed as standalone products; instead, every specific vaccine formulation containing an adjuvant is evaluated as a new biological entity, requiring tailored non-clinical toxicology (including local reactogenicity and biodistribution), dose-finding, and large comparative safety studies before licensure. Long-term safety evaluation focuses on post-marketing safety analyses and the potential to trigger or exacerbate autoimmune diseases (autoimmune/inflammatory syndrome induced by adjuvants; ASIA). These conservative guidelines contribute to the current situation, with many novel adjuvants having been tested in clinical trials, but only a few show sufficient benefit–risk profiles for approval [87,88,89,90].

## 7. Outlook—Anthralin as a Platform Power Adjuvant

While anthralin has been successfully combined with IMQ as a co-adjuvant in TCI, pairing it with alternative pattern recognition receptor (PRR) agonists could further enhance immune responses by targeting complementary innate sensing pathways. The STING (Stimulator of Interferon Genes) agonist, for instance, represents a promising candidate due to its proven potency in immunization settings, yet TCI applications remain unexplored [91,92,93]. Unlike TLR7/8 agonists such as IMQ, STING agonists activate cytosolic DNA sensing, potentially synergizing with anthralin’s ROS-dependent monocyte recruitment to achieve superior immune responses. Other candidates such as TLR9 agonists (CpG ODNs), RIG-I agonists (Poly-ICLC), or even low-dose TLR4 agonists (MPL/GLA) could similarly complement anthralin’s unique inflammatory niche and could be explored [94,95,96].

Triple or even quadruple adjuvant combinations represent an exciting frontier for TCI optimization. Anthralin primes an inflammatory milieu in skin that is optimally suitable for layering PRR agonists targeting distinct subcellular compartments. These combinations can theoretically maximize innate amplification while minimizing individual dose requirements, though skin tolerability and cytokine storm risks require careful optimization.

Combination therapy with systemic immune checkpoint inhibitors offers another opportunity. While α-PD(L)-1 and ipilimumab demonstrate clinical efficacy in melanoma and other cancers, their incomplete tumor rejection rates and serious side effects limit their benefit [77,97,98]. Anthralin-based TCI platforms such as DIVA have the potential to generate synergistic effects with immune checkpoint inhibitors and consequently improve treatment success.

## 8. Conclusions

Anthralin has evolved from a classical antipsoriatic agent into a promising topical adjuvant, and the data reviewed here suggest that its pro-inflammatory effects can be harnessed to shape a local immune environment that supports antigen uptake, myeloid cell recruitment, and cytotoxic T-cell priming. In combination with IMQ, anthralin has enabled highly potent DIVA responses in mouse models, including strong CTL induction, memory formation, and prophylactic protection in tumor and viral challenge settings. These findings make anthralin an attractive candidate for transcutaneous immunization, particularly where durable cellular immunity is desired.

At the same time, the translational path remains clearly defined by several open questions. Most available data have been generated in healthy mouse skin, whereas human application will need to account for differences in skin structure, follicular architecture, barrier function, cytokine networks, and tissue-specific immune regulation. It also remains uncertain how anthralin will perform in patients with inflamed, barrier-disrupted, or otherwise diseased skin, where baseline immune activation may either amplify or distort the intended vaccine effect. Likewise, the optimal treated area, dose, antigen format, and peptide penetration strategy for humans are still unknown.

Taken together, anthralin represents a compelling example of how a long-used dermatologic drug may be repurposed into an immunological tool. Its future as a vaccine adjuvant will depend on whether its local inflammatory activity can be tuned to reliably promote APC activation and T-cell priming without excessive reactogenicity. If these translational hurdles can be addressed, anthralin-based formulations could provide a practical route toward needle-free transcutaneous immunization strategies for infectious disease and cancer.

## Data Availability

No new data were created or analyzed in this study. Data sharing is not applicable to this article.
